# High-pressure synthesis and electrochemical properties of tetragonal LiMnO_2_†

**DOI:** 10.1039/c8ra03722a

**Published:** 2018-07-24

**Authors:** Takeshi Uyama, Kazuhiko Mukai, Ikuya Yamada

**Affiliations:** Toyota Central Research and Development Laboratories, Inc. Nagakute Aichi 480-1192 Japan e1599@mosk.tytlabs.co.jp; Department of Materials Science, Graduate School of Engineering, Osaka Prefecture University 1-2 Gakuen Sakai Osaka 599-8590 Japan

## Abstract

Tetragonal structured LiMnO_2_ (t-LiMnO_2_) samples were synthesized under pressures above 8 GPa and investigated as a positive electrode material for lithium-ion batteries. Rietveld analyses based on X-ray diffraction measurements indicated that t-LiMnO_2_ belongs to a γ-LiFeO_2_-type crystal structure with the *I*4_1_/*amd* space group. The charge capacity during the initial cycle was 37 mA h g^−1^ at 25 °C, but improved to 185 mA h g^−1^ at 40 °C with an average voltage of 4.56 V *vs.* Li^+^/Li. This demonstrated the superiority of t-LiMnO_2_ over other lithium manganese oxides in terms of energy density. The X-ray diffraction measurements and Raman spectroscopy of cycled t-LiMnO_2_ indicated an irreversible transformation from the γ-LiFeO_2_-type structure into a Li_*x*_Mn_2_O_4_ spinel structure by the displacement of 25% of the Mn ions to vacant octahedral sites through adjacent octahedral sites.

## Abstract 2

Tetragonal structured LiMnO_2_ (t-LiMnO_2_) samples were synthesized under pressures above 8 GPa and investigated as a positive electrode material for lithium-ion batteries.

## Graphical abstract

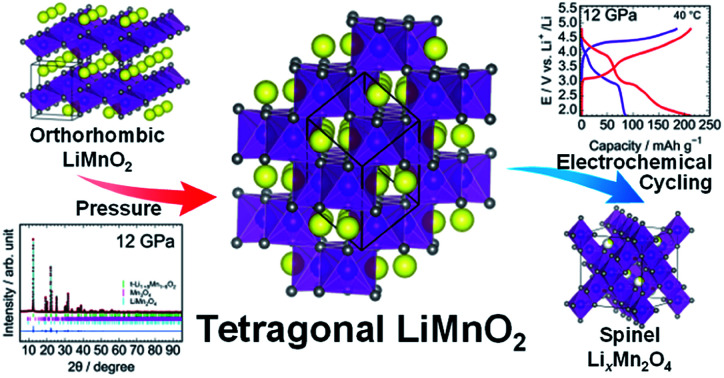

## Introduction

**Fig. 1:**
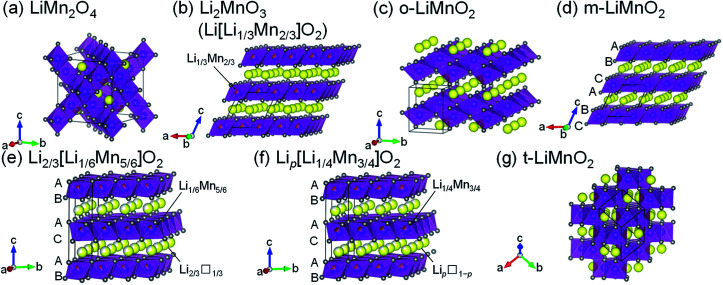
Crystal structures of seven lithium manganese oxides: (a) LiMn_2_O_4_, (b) Li_2_MnO_3_ (Li[Li_1/3_Mn_2/3_]O_2_), (c) o-LiMnO_2_, (d) m-LiMnO_2_, (e) Li_2/3_[Li_1/6_Mn_5/6_]O_2_, (f) Li_*p*_[Li_1/4_Mn_3/4_]O_2_, and (g) t-LiMnO_2_. Yellow, purple, and gray spheres indicate Li, Mn, and O atoms, respectively. Unit cells are shown by the solid black lines. The oxygen stacking manner is shown in Fig. 1d–f.

Lithium manganese oxides (LMOs) have been extensively studied as a potential positive electrode material for lithium-ion batteries (LIBs), due to their low cost and environmental friendliness.^1–6^ Since such properties are crucial for large-scale applications for LIBs such as electric vehicles and grid energy storage systems,^7^ research efforts have been devoted to the development of LMOs with high energy densities (*W*_s_). Table 1 summarizes the structure types, synthesis methods, and electrochemical properties of previously reported LMOs, and their crystal structures are shown issn Fig. 1a–g. Of these LMOs, a cubic spinel-structured LiMn_2_O_4_ (Fig. 1a) is widely accepted as the most practical positive electrode material.^1,2,8^ LiMn_2_O_4_ exhibits a rechargeable capacity (*Q*_recha_) of approximately 120 mA h g^−1^ with an average voltage (*E*_ave_) of 4.1 V *vs.* Li^+^/Li, and its *W* approaches the theoretical limit value due to the moderate theoretical capacity (*Q*_theo_) of 148 mA h g^−1^.

**Table 1:** Crystal structures, synthesis methods, and electrochemical properties of various lithium manganese oxides

Compounds	Structure types (space group)	Synthesis methods	*Q*_theo_ a/mA h g^−1^	Electrochemical properties	Remarks	References
LiMn_2_O_4_	Cubic spinel (*Fd*3̄*m*)	Solid-state reaction	148 (*n* = 1)	*Q*_recha_ ∼ 120 mA h g^−1^, *E*_ave_ ∼ 4.1 V		1, 2 and 8
Li_2_MnO_3_ (Li[Li_1/3_Mn_2/3_]O_2_)	Monoclinic layer (*C*2/*m*)	Solid-state reaction	459 (*n* = 2)	*Q*_recha_ ∼ 0 mA h g^−1^	Electrochemically inactive	9 and 10
o-LiMnO_2_	β-NaMnO_2_ (*Pmmn*)	Solid-state reaction	285 (*n* = 1)	*Q*_cha_ = 180–230 mA h g^−1^, *E*_ave_ ∼3.6 V (first charge only)	Transformation into spinel phase on the charge reaction	12–18
m-LiMnO_2_	α-NaMnO_2_ (*C*2/*m*)	Ion exchange method	285 (*n* = 1)	*Q*_cha_ = 270 mA h g^−1^, *E*_ave_ ∼ 3.8 V (first charge only)	Transformation into spinel phase on the charge reaction	4 and 19
Li_2/3_[Li_1/6_Mn_5/6_]O_2_	O2-type layer (*P*3*m*1)	Ion exchange method	193 (*n* = 2/3)	*Q*_recha_ = 150 mA h g^−1^, *E*_ave_ ∼ 3.2 V	Decrease of the *Q*_recha_ in a full cell	20
Li_*p*_[Li_1/4_Mn_3/4_]O_2_ (*p* < 1)	O2-type layer (*P*6_3_*mc*)	Ion exchange method	<327 (*n* < 1)	*Q*_recha_ = 200 mA h g^−1^, *E*_ave_ ∼ 3.2 V	Decrease of the *Q*_recha_ in a full cell	21
t-LiMnO_2_	γ-LiFeO_2_ (*I*4_1_/*amd*)	High-pressure and high-temperature method	285 (*n* = 1)	Unknown		This work

a Theoretical capacity (*Q*_theo_) is calculated assuming that a charge-transfer number is *n*.

In terms of the *Q*_theo_, LiMnO_2_ and its derivatives are appealing, and their structural and electrochemical properties vary depending on their composition and method of synthesis. A monoclinic layered Li_2_MnO_3_, also written as Li[Li_1/3_Mn_2/3_]O_2_ (Fig. 1b), has a large *Q*_theo_ of 458 mA h g^−1^. However, Li_2_MnO_3_ is essentially electrochemically inactive because of the difficulty in further oxidizing the Mn^4+^ species,^9^ unless a proton exchange^10^ and/or an oxygen loss^11^ in its lattice proceed during the initial charge reaction. An orthorhombic LiMnO_2_ (o-LiMnO_2_)^12–18^ and a monoclinic LiMnO_2_ (m-LiMnO_2_)^4,19^ have a formal oxidation state of Mn^3+^ (Fig. 1c and d) and their charge capacities (*Q*_cha_s) reach a maximum of 230 mA h g^−1^ for o-LiMnO_2_ and 270 mA h g^−1^ for m-LiMnO_2_. However, delithiated o- and m-LiMnO_2_ can be irreversibly and spontaneously transformed into a Li_*x*_Mn_2_O_4_ spinel; thus, the *Q*_recha_ values for both of these compounds decrease in a manner similar to that seen in LiMn_2_O_4_ as the changes in the charge and discharge curves echo those of Li_*x*_Mn_2_O_4_.^12,15–19^ In contrast, O2-type layered Li_2/3_[Li_1/6_Mn_5/6_]O_2_ (ref. 20) and Li_*p*_[Li_1/4_Mn_3/4_]O_2_ with *p* < 1 (ref. 21) (Fig. 1e and f) were reported to display good cycleability without transformation into the Li_*x*_Mn_2_O_4_ spinel. The oxygen stacking, which is ABACAB for Li_2/3_[Li_1/6_Mn_5/6_]O_2_ and Li_*p*_[Li_1/4_Mn_3/4_]O_2_ (unlike the ABCABC stacking seen in m-LiMnO_2_), plays an important role in this. As a result, the *Q*_recha_ is 150 mA h g^−1^ for Li_2/3_[Li_1/6_Mn_5/6_]O_2_ and 200 mA h g^−1^ for Li_*p*_[Li_1/4_Mn_3/4_]O_2_. In the initial cycle, the *Q*_cha_ ranges between 20 and 50 mA h g^−1^, and is smaller than the *Q*_recha_ due to lithium deficiency in these compounds. This triggers a decrease in the *Q*_recha_ whenever these compounds are used in full cells with negative electrode materials that do not contain residual lithium, *e.g.* graphite and silicon.

Sugiyama *et al.* synthesized a tetragonal LiMnO_2_ (t-LiMnO_2_) from o-LiMnO_2_ by a high-pressure and high-temperature method, with pressures between 4 and 6 GPa and temperatures between 900 and 1200 °C.^22^ Rietveld analyses based on X-ray diffraction (XRD) measurements showed that t-LiMnO_2_ has a γ-LiFeO_2_-type structure with *I*4_1_/*amd* space group (Fig. 1g). In this structure, MnO_6_ octahedra form a three-dimensional framework by sharing their edges, resulting in a straight channel of Li^+^ ions along the [111] direction. This configuration between MnO_6_ octahedra and Li^+^ ions is clearly different from the framework composed of two-dimensional MnO_6_ layers in the LiMnO_2_-based compounds mentioned above (see Fig. 1b–f); rather, the crystal structure of t-LiMnO_2_ is comparable to that of LiMn_2_O_4_. Therefore, in addition to the high *Q*_theo_ (285 mA h g^−1^), t-LiMnO_2_ is also a desirable material for understanding the relationships between the structural and electrochemical properties in a group of LMOs.

Despite its unique crystallographic character, the electrochemical properties of t-LiMnO_2_ remain unclear. This is likely due to the difficulty in conducting large-scale synthesis *via* the high-pressure method because a tiny sample container is generally used to generate high pressure (>10 GPa).^23^ However, recent technological developments, including the use of belt-type and multi-anvil-type equipment, enabled us to evaluate a variety of functional materials.^24–27^ In the current study, we synthesized t-LiMnO_2_ under high pressures up to 12 GPa and reported its electrochemical performance as a positive electrode material for LIBs for the first time, in order to clarify the relationship between the structural and electrochemical properties of t-LiMnO_2_. The *Q*_cha_ of t-LiMnO_2_ reached 185 mA h g^−1^ with an *E*_ave_ of 4.56 V upon increasing the operating temperature to 40 °C, which is superior to other LMOs. This information will be helpful in designing advanced LMOs with high *W* in terms of their structure and composition.

## Experimental

### Sample preparations

t-LiMnO_2_ was synthesized from o-LiMnO_2_ by the high-pressure and high-temperature method using a Walker-type apparatus.^28^ o-LiMnO_2_ was first synthesized *via* solid-state reactions. Stoichiometric amounts of LiOH·H_2_O (Wako Pure Chemical Industries Ltd.) and Mn_2_O_3_ (Wako Pure Chemical Industries Ltd.) were mixed in ethanol and the mixture was dried and pressed into a 5 mm-thick pellet with a diameter of 15 mm. The pellet was heated at 1000 °C for 12 h under argon gas flow at a heating rate of 200 °C h^−1^ and a cooling rate of 1 °C min^−1^. The obtained o-LiMnO_2_ was crushed and re-pressed into a 5 mm-thick pellet with a diameter of 2.8 mm before being packed into a platinum capsule. The capsule was then placed in a BN insulation sleeve, which was placed in a cylindrical graphite heater. The assembled sample was placed in a (Mg, Co)O octahedral pressure medium with side lengths of 14 mm. The (Mg, Co)O octahedra were slowly compressed to 5, 8, or 12 GPa by eight tungsten carbide truncated 8 mm edges. The compressed samples were then heated at 1000 °C for 30 min and subsequently quenched to room temperature, and the pressure was slowly released until an ambient pressure was achieved. Hereafter, the o-LiMnO_2_ samples treated at 5, 8, and 12 GPa are represented as LMO (5 GPa), LMO (8 GPa), and LMO (12 GPa), respectively, to avoid a misunderstanding of the actual phase purity. A powder sample of LiMn_2_O_4_ was also synthesized by heating a mixture of LiOH·H_2_O and MnO_2_ (Kojundo Chemical Laboratory Co., Ltd.) at 1000 °C for 12 h under oxygen gas flow.

### Characterization of the samples

The powder samples of o-LiMnO_2_, LMO (5 GPa), LMO (8 GPa), and LMO (12 GPa) were characterized by scanning electron microscopy (SEM), synchrotron XRD measurements, and Raman spectroscopy. The SEM images were recorded using an S-3600N (Hitachi High-Technologies, Company Ltd.) at an accelerating voltage of 15 kV. The XRD measurements were conducted at a BL5S2 beamline available at the Aichi Synchrotron Radiation Center. The samples were packed into borosilicate glass capillary tubes with a diameter of 0.3 mm (WJM-Glas Müller GmbH). The XRD patterns were collected using a two-dimensional detector, PILATUS 100K (Dectris Ltd., Baden-Daettwil), over a 2*θ* range between 5° and 95°. The incident X-ray wavelength (*λ*) was determined to be 0.799323(2) or 0.799670(2) Å from the XRD patterns of silicon powders (NIST 640d). Rietveld analyses and drawings of crystal structures were carried out using the RIETAN-FP^29^ and VESTA softwares,^30^ respectively. The Raman spectra were collected on an NRS-3300 (Jasco Co. Ltd.) using an excitation laser wavelength of 532 nm and a laser power of 0.1 mW. The duration of exposure was 600 s.

### Electrochemical properties

The electrochemical reactivities of the o-LiMnO_2_, LMO (5 GPa), LMO (8 GPa), and LMO (12 GPa) samples were examined with sandwich-type two-electrode cells. The powdered sample, acetylene black (AB, Denka Co., Ltd.), and polytetrafluoroethylene (PTFE, Daikin Industries Ltd.) were combined in a ratio of 70 : 20 : 10 to give a viscoelastic mixture, which was then pressed onto an aluminum mesh current collector with a diameter of 16 mm. The mixture functioned as a working electrode. Lithium metal pressed onto 19 mm-wide stainless steel was used as a counter electrode. The electrolyte was made from a solution of 1 M LiPF_6_ dissolved in ethylene carbonate (EC)/dimethyl carbonate (DMC) at an EC : DMC ratio of 3 : 7 v/v (Kishida Chemical Company Ltd.) and the separator was constructed from two sheets of porous polyethylene membrane (Tonen-General Sekiyu K. K.). The cells were assembled in an argon-filled glove box and operated at a current density of 0.025 mA cm^−2^. The voltage ranged from 1.8 to 4.8 V and the operating temperature was set at 25 or 40 °C.

After cycling test at 25 °C, crystal structures of the samples were investigated using synchrotron XRD and Raman measurements to clarify both macroscopic and microscopic structural changes during the cycling. The cells were charged and discharged twenty times at 25 °C. The cycled samples were then taken from the working electrodes, which had been thoroughly rinsed with a diethyl carbonate solution. Each of the samples was packed into a capillary tube with a diameter of 0.7 mm and put into a quartz glass cell (GL Sciences Inc.) for the XRD measurements and for the Raman spectroscopy, respectively. All the procedures were conducted in the argon-filled glove box, so as not to contact with the atmosphere. The laser power of the Raman spectroscopy was set at 1.0 mW in consideration of absorbance of the quartz cell. The Raman spectra of the charged LMO (12 GPa) sample, LiMn_2_O_4_, PTFE, and AB were also measured.

## Results and discussion

### Morphological and structural characterization

**Fig. 2:**
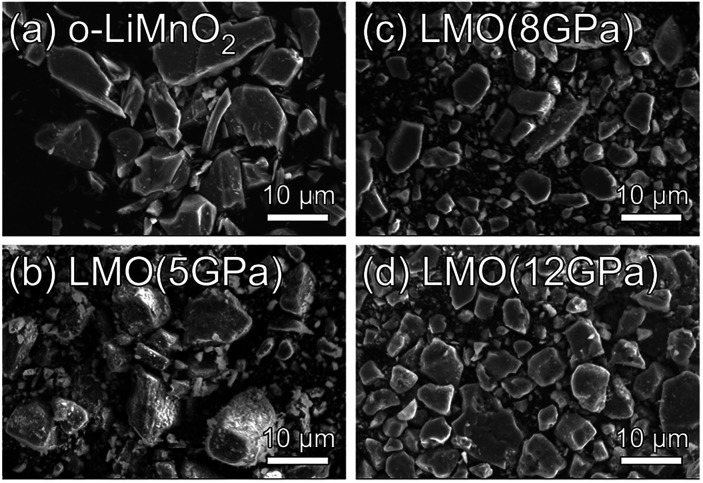
SEM images for the (a) o-LiMnO_2_, (b) LMO (5 GPa), (c) LMO (8 GPa), and (d) LMO (12 GPa) samples.

Fig. 2a–d show SEM images for the o-LiMnO_2_, LMO (5 GPa), LMO (8 GPa), and LMO (12 GPa) samples, respectively. Particles of o-LiMnO_2_ exhibit flake-like shapes with widths between 2–10 μm and thicknesses less than 2 μm. The particle shapes appear to round out and become uniform in size with increasing applied pressure, *i.e.*, rough shapes with a dispersive size of 1–10 μm at 5 GPa and smooth morphologies with a dominant size of ∼8 μm at 8 GPa and 12 GPa were observed. This morphological change is likely due to a phase transformation, as will be discussed below.

**Fig. 3:**
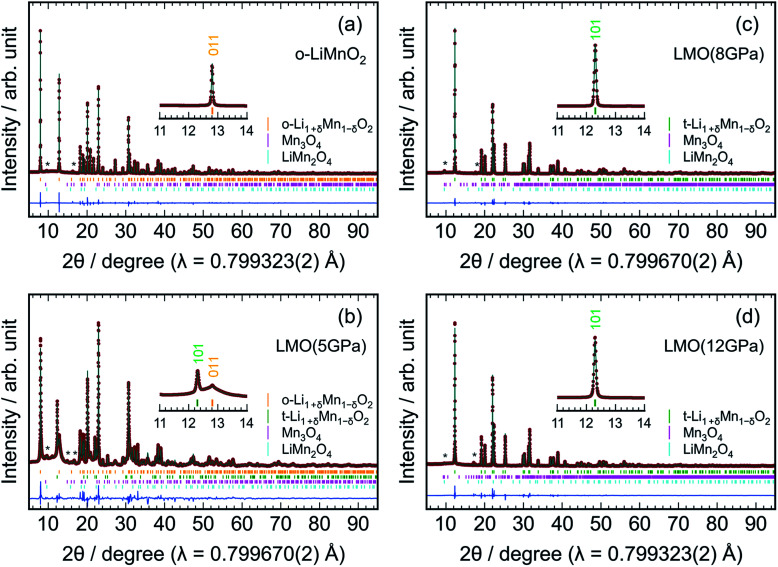
Rietveld results for the (a) o-LiMnO_2_, (b) LMO (5 GPa), (c) LMO (8 GPa), and (d) LMO (12 GPa) samples. Enlarged patterns between 11° and 14° are also shown in the insets to clarify a phase transformation. The orange, green, magenta, and cyan vertical lines indicate the Bragg positions for orthorhombic Li_1+*δ*_Mn_1−*δ*_O_2_ with *Pmmn* space group, tetragonal Li_1+*δ*_Mn_1−*δ*_O_2_ with *I*4_1_/*amd*, Mn_3_O_4_ with *I*4_1_/*amd* or *Pbcm* space group, and LiMn_2_O_4_ with *Fd*3̄*m* space group, respectively. The diffraction lines from the Mn_3_O_4_ and LiMn_2_O_4_ impurities are denoted by asterisks (*). Red dots and solid green lines indicate observed and calculated profiles, respectively, whereas solid blue lines show the differences between the observed and the calculated profiles.

Fig. 3 shows the results of Rietveld analyses for the (a) o-LiMnO_2_, (b) LMO (5 GPa), (c) LMO (8 GPa), and (d) LMO (12 GPa) samples. The crystal structure of the o-LiMnO_2_ sample was assigned as an orthorhombic β-NaMnO_2_-type structure with *Pmmn* space group; that is, a zigzag layered structure in which Li and Mn atoms occupy each of the octahedral 2*b* sites (Fig. 1c). However, the o-LiMnO_2_ sample is not in a single phase but contained some impurity phases as shown by asterisk marks at around 2*θ* = 10° and 16°, and these were found to be hausmannite (Mn_3_O_4_ with *I*4_1_/*amd* space group) and spinel LiMn_2_O_4_ by examining the possible crystal structures of LMOs and manganese oxides. Thus, we performed Rietveld analyses with three coexisting phases, in which a Mn under-stoichiometric Li_1+*δ*_Mn_1−*δ*_O_2_ is adopted to refine the crystal structure of o-LiMnO_2_. Here, *δ* represents the Mn deficiency due to impurities. Moreover, we considered Li and Mn atoms to randomly occupy each of the 2*b* sites, as a cation mixing of Li and transition metal ions occasionally occurs in lithium insertion materials.^31^ The structure parameters of the o-LiMnO_2_ sample are listed in Table 2. The lattice parameters of o-LiMnO_2_ were calculated to be *a*_o_ = 2.80700(2) Å, *b*_o_ = 4.57721(2) Å, and *c*_o_ = 5.75210(3) Å. The actual composition was determined to be Li_1.005_Mn_0.995_O_2_ (*δ* = 0.005), where 1.5% of the Mn ions occupied Li (2*b*) sites. The weight fractions were 98.9 wt% for Li_1.005_Mn_0.995_O_2_, 0.7 wt% for Mn_3_O_4_, and 0.4 wt% for LiMn_2_O_4_, giving a Li/Mn ratio of 0.999/1.000, which was consistent with the Li/Mn ratio of the starting material.

**Table 2:** Structure parameters of the o-LiMnO_2_ sample determined by Rietveld analyses

Phase	Space group	Atom	Wyckoff position	*g* a	*x*	*y*	*z* a	*B* a/Å^2^
o-LiMnO_2_	*Pmmn*	Li1	2*b*	0.985(1)	1/4	3/4	0.1188(9)	1.32(8)
		Mn1	2*b*	0.015(1)	1/4	3/4	0.1188(9)	1.32(8)
		Mn2	2*b*	0.980(1)	1/4	3/4	0.6347(1)	0.55(1)
		Li2	2*b*	0.020(1)	1/4	3/4	0.6347(1)	0.55(1)
		O1	2*a*	1	1/4	1/4	0.1432(3)	0.58(2)
		O2	2*a*	1	1/4	1/4	0.6004(3)	0.58(2)
Composition: Li_1.005_Mn_0.995_O_2_ (*δ* = 0.005), *a*_o_ = 2.80700(2) Å, *b*_o_ = 4.57721(2) Å, and *c*_o_ = 5.75210(3) Å								
Reliable factors: *R*_wp_ = 5.658%, *R*_p_ = 4.184%, and *S* = 0.4501								
Mass fractions: 98.9 wt% for o-LiMnO_2_, 0.7 wt% for Mn_3_O_4_, and 0.4 wt% for LiMn_2_O_4_								

a Constraints: *g*(Mn1) = 1 − *g*(Li1), *g*(Mn2) = *g*(Li1) − *δ*, *g*(Li2) = 1 + *δ* − *g*(Li1), *z*(Mn1) = *z*(Li1), *z*(Li2) = *z*(Mn2), *B*(Mn1) = *B*(Li1), *B*(Li2) = *B*(Mn2), and *B*(O2) = *B*(O1).

For the LMO (5 GPa) sample, the diffraction line at 2*θ* = 12.8° decreases, but the diffraction line at 2*θ* = 12.3° clearly generates, due to the formation of the t-LiMnO_2_ phase (see the inset of Fig. 3b). As listed in Table S1,† the mass fractions of o-LiMnO_2_ and t-LiMnO_2_ were found to be 86.6 and 8.6 wt%, respectively. For the LMO (8 GPa) and LMO (12 GPa) samples, the diffraction line at 2*θ* = 12.8° disappears, and almost all diffraction lines can be assigned as the tetragonal γ-LiFeO_2_-type structure with *I*4_1_/*amd* space group (Fig. 3c and d). The hausmannite impurity also transformed into a CaMn_2_O_4_-type structure (*Pbcm*) with an applied pressure up to 8 GPa.^32^Table 3 shows the structure parameters of the LMO (12 GPa) sample as determined by Rietveld analyses under identical assumptions as the o-LiMnO_2_ sample. The tetragonal lattice parameters, *a*_t_ and *c*_t_, were determined to be 4.18278(2) and 8.22922(6) Å, respectively. Note that the actual composition of t-LiMnO_2_, Li_1.012_Mn_0.988_O_2_ (*δ* = 0.012), was almost identical to that of o-LiMnO_2_. From the occupancy factor, *g*, it was shown that Mn ions occupy 0.8% of the Li (4*a*) sites. As shown in Table S3,† there were no significant differences in the structural parameters, including the *a*_t_, *c*_t_, composition, atomic coordination, and *g* between the LMO (8 GPa) and LMO (12 GPa) samples.

**Table 3:** Structure parameters of the LMO (12 GPa) sample determined by Rietveld analyses

Phase	Space group	Atom	Wyckoff position	*g* a	*x*	*y*	*z*	*B* a/Å^2^
t-LiMnO_2_	*I*4_1_/*amd*	Li1	4*a*	0.992(1)	0	3/4	1/8	1.31(9)
		Mn1	4*a*	0.008(1)	0	3/4	1/8	1.31(9)
		Mn2	4*b*	0.980(1)	0	1/4	3/8	0.51(1)
		Li2	4*b*	0.020(1)	0	1/4	3/8	0.51(1)
		O1	8*e*	1	0	1/4	0.1413(1)	1.2(2)
Composition: Li_1.012_Mn_0.988_O_2_ (*δ* = 0.012), *a*_t_ = 4.18278(2) Å, and *c*_t_ = 8.22922(6) Å								
Reliable factors: *R*_wp_ = 6.403%, *R*_p_ = 4.544%, and *S* = 0.4712								
Mass fractions: 97.8 wt% for t-LiMnO_2_, 1.8 wt% for Mn_3_O_4_, and 0.4 wt% for LiMn_2_O_4_								

a Constraints: *g*(Mn1) = 1 − *g*(Li1), *g*(Mn2) = *g*(Li1) − *δ*, *g*(Li2) = 1 + *δ* − *g*(Li1), *B*(Mn1) = *B*(Li1), and *B*(Li2) = *B*(Mn2).

The change in the XRD patterns indicated that o-LiMnO_2_ gradually transformed into t-LiMnO_2_ at 5 GPa, with the phase transformation occurring as the pressure reached 8 GPa. Conversely, Sugiyama *et al.* reported that this phase transformation was completed at a pressure of 5 GPa in Mn over-stoichiometric Li_0.93_Mn_1.07_O_2_.^22^ Therefore, the transformation from o-LiMnO_2_ to t-LiMnO_2_ is very sensitive to the Li/Mn ratio of o-LiMnO_2_; Mn under-stoichiometric Li_1.1_Mn_0.9_O_2_ (*δ* = 0.1) actually transformed into a rock-salt structure rather than into t-LiMnO_2_ at 5 GPa and 1000 °C.^22^

**Fig. 4:**
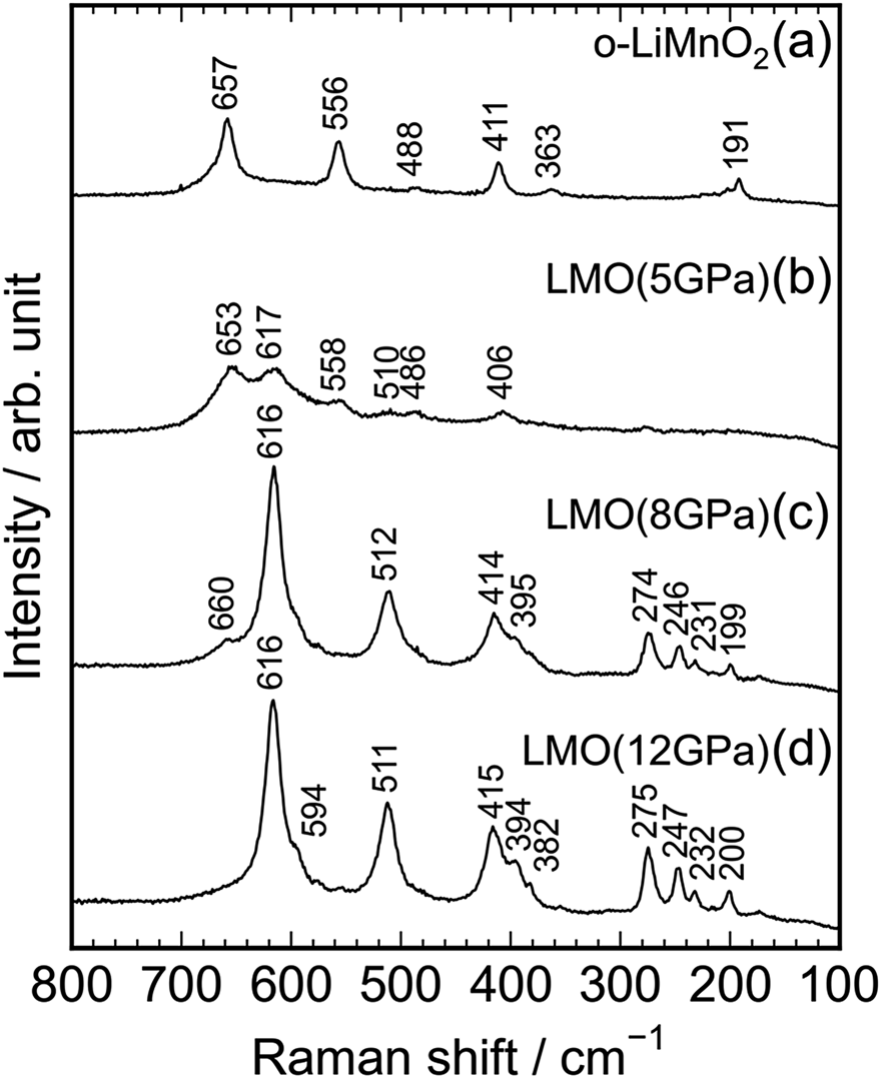
Raman spectra of the (a) o-LiMnO_2_, (b) LMO (5 GPa), (c) LMO (8 GPa), and (d) LMO (12 GPa) samples.

Fig. 4a–d show Raman spectra of the o-LiMnO_2_, LMO (5 GPa), LMO (8 GPa), and LMO (12 GPa) samples, respectively. Factor group analyses^33^ predicted twelve Raman bands of 4*A*_g_ + 4*B*_2g_ + 4*B*_3g_ for o-LiMnO_2_ structure and eight Raman bands of *A*_1g_ + 3*B*_1g_ + 4*E*_g_ for t-LiMnO_2_ structure. The Raman spectrum for the o-LiMnO_2_ sample shows three major Raman bands at 411, 556, and 657 cm^−1^ and three minor Raman bands at 191, 363, and 488 cm^−1^. Although all of the Raman bands are not fully assigned, according to the previous reports,^34–36^ the major Raman bands are attributed to vibrations in the MnO_6_ octahedra, that is, the O–Mn–O stretching modes at 657 cm^−1^, the O–Mn–O bending modes at 556 cm^−1^, and the Mn–O–Mn deformation modes at 441 cm^−1^. The Raman spectrum of the LMO (5 GPa) sample is similar with that of the o-LiMnO_2_ sample, however, the intensities of the Raman bands at ∼411, 556, and 657 cm^−1^ become weak compared with its original Raman spectrum. Furthermore, new and weak Raman bands are observed at 406, 510, and 617 cm^−1^, and these Raman bands are clearly observed in the Raman spectra of the LMO (8 GPa) and LMO (12 GPa) samples. There are at least ten Raman bands in the Raman spectrum of the LMO (12 GPa) sample, although the theoretical calculation predicted eight Raman bands. This difference is probably due to the nonstoichiometry and/or impurity phases in the LMO (12 GPa) sample. Anyway Raman spectroscopy clarified that the transformation from o-LiMnO_2_ to t-LiMnO_2_ is achieved under pressures above 8 GPa, which is consistent with the results of the XRD measurements.

### Electrochemical properties

**Fig. 5:**
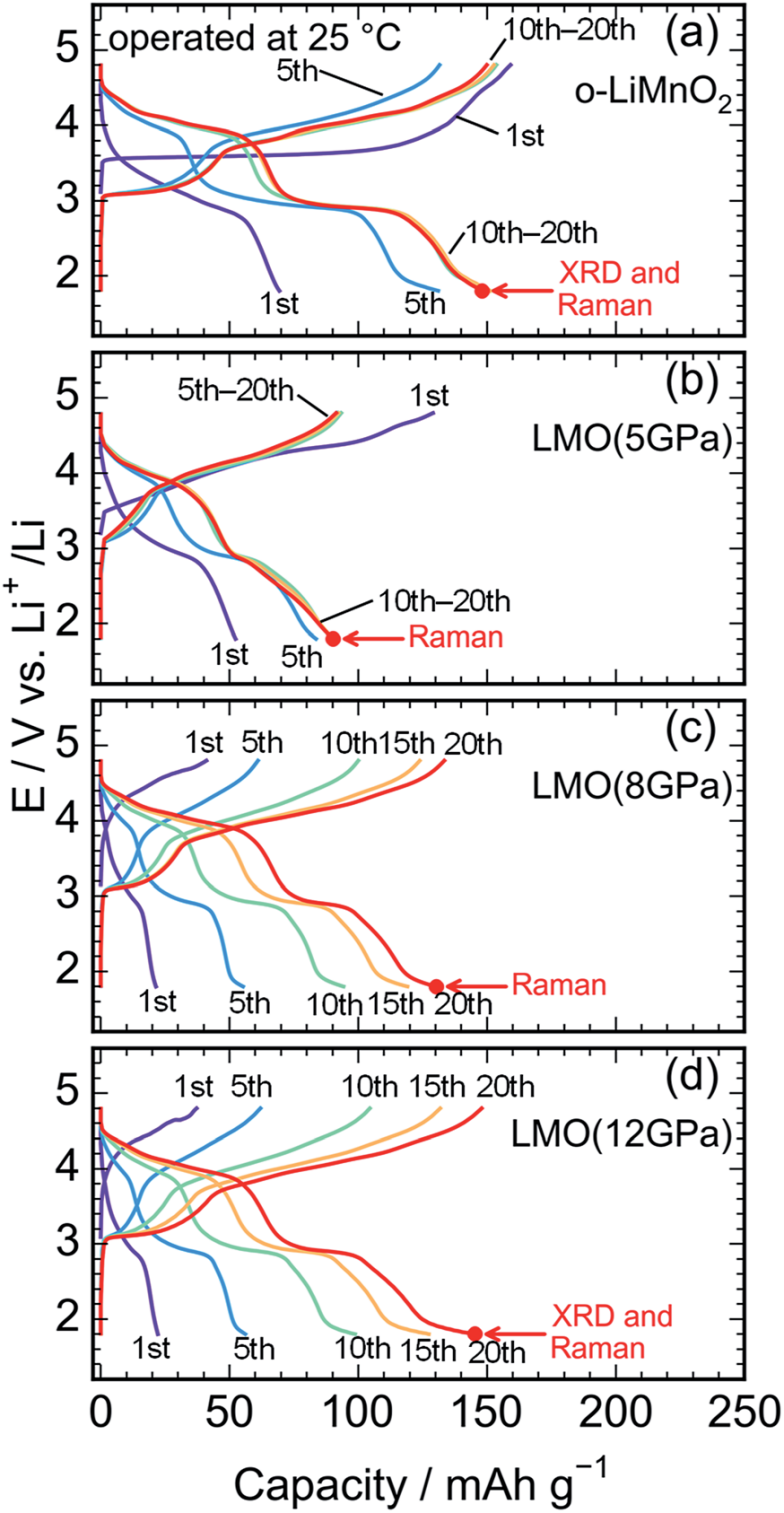
Charge and discharge curves of the lithium cells with the (a) o-LiMnO_2_, (b) LMO (5 GPa), (c) LMO (8 GPa), and (d) LMO (12 GPa) samples. The cells were operated at temperature of 25 °C. *Ex situ* XRD measurements and Raman spectroscopy were conducted at the discharged state, as indicated by the red arrows.

Fig. 5a shows charge and discharge curves of the Li cell with the LiMnO_2_ sample, operated at temperature of 25 °C. The cell voltage (*E*) increases rapidly from an open circuit voltage (∼3.1 V) before plateauing at ∼3.5 V when the *Q*_cha_ reaches 120 mA h g^−1^. Then, the *E* climbs to 4.8 V with a gentle gradient during the initial charge. In the discharge curve, however, the *E* drops sharply to 3.5 V, and gradually decreases to ∼3.0 V without any clear voltage plateaus. The *Q*_cha_ is 160 mA h g^−1^, whereas discharge capacity (*Q*_dis_) is only 70 mA h g^−1^. New voltage plateaus appeared at ∼3.0 and 4.0 V in subsequent charge and discharge curves. The differences in charge and discharge curves between 1^st^ and subsequent cycles is caused by an irreversible structural transformation to the Li_*x*_Mn_2_O_4_ spinel during the initial cycle, as previously reported.^12,18,37^

Fig. 5b–d display the charge and discharge curves of the lithium cells with the LMO (5 GPa), LMO (8 GPa), and LMO (12 GPa) samples, respectively. The charge and discharge curves of these three samples are clearly different from those of o-LiMnO_2_; *i.e.*, the *E* monotonically increases from 3.2 V to 4.8 V without any apparent voltage plateaus. The *Q*_cha_ of LMO (5 GPa) is 154 mA h g^−1^, while the *Q*_cha_ values of LMO (8 GPa) and LMO (12 GPa) are 41 and 37 mA h g^−1^, respectively, which are approximately 13% of the *Q*_theo_. The initial discharge curves have a similar *E* profile with a broad center at ∼3.0 V. The *Q*_dis_ values of the LMO (5 GPa), LMO (8 GPa), and LMO (12 GPa) samples are 52, 21, and 22 mA h g^−1^, respectively. Besides the decline in the *E*, as similarly noted for o-LiMnO_2_, both the charge and discharge curves display multiple voltage plateaus with an increasing cycle number. This implied that t-LiMnO_2_ irreversibly transformed into the Li_*x*_Mn_2_O_4_ spinel with the electrochemical cycling. The cycle performances of these four samples are given in Fig. S1.† The *Q*_cha_ and *Q*_dis_ values of o-LiMnO_2_ [LMO (5 GPa)] increase with the cycle numbers until tenth cycles, and then maintain at ∼150 (100) mA h g^−1^. On the other hand, the *Q*_cha_ and *Q*_dis_ values of LMO (8 GPa) and LMO (12 GPa) increase with the cycle numbers up to twenty cycles. The *Q*_cha_ and *Q*_dis_ values of LMO (12 GPa) are similar with those of o-LiMnO_2_.

**Fig. 6:**
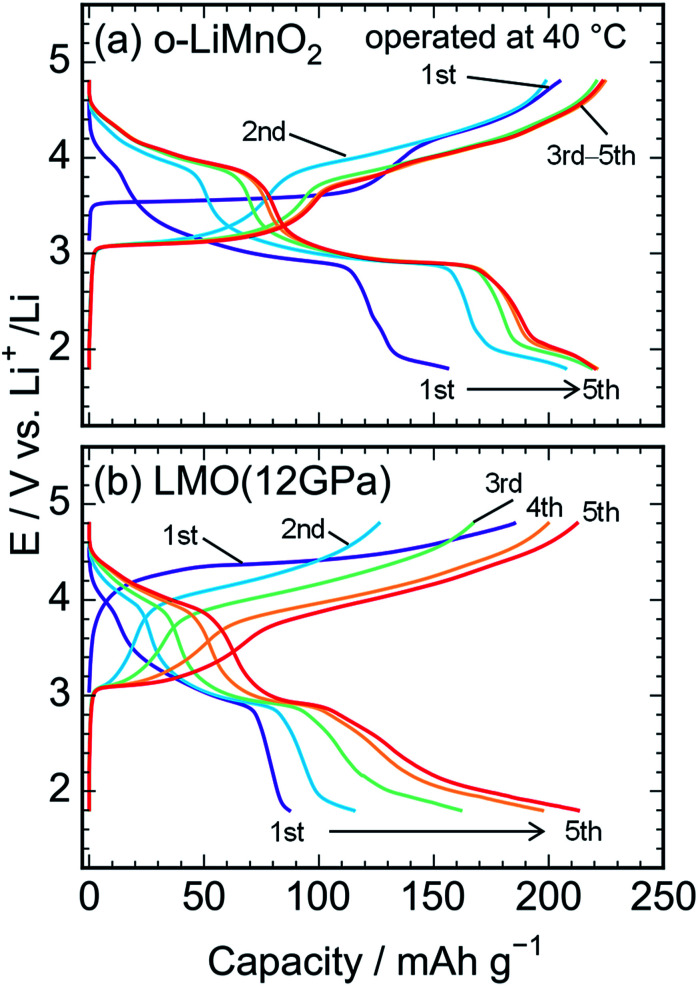
Charge and discharge curves of the lithium cells with the (a) o-LiMnO_2_ and (b) LMO (12 GPa) samples. The cells were operated at temperature of 40 °C.

Fig. 6a and b show charge and discharge curves of the lithium cells with the o-LiMnO_2_ and LMO (12 GPa) samples, respectively, operated at temperature of 40 °C. The initial *Q*_cha_ of o-LiMnO_2_ is approximately 200 mA h g^−1^, which is larger than the value (=160 mA h g^−1^) obtained at 25 °C. This originates from the appearance of a moderate voltage plateau over 4.0 V with a *Q*_cha_ of ∼50 mA h g^−1^, as reported for an o-LiMnO_2_/Li cell at 55 °C by Cho.^38^ Subsequent charge and discharge curves reveal the increases in *Q*_cha_ from 200 mA h g^−1^ to 225 mA h g^−1^ and in *Q*_dis_ from 155 mA h g^−1^ to 220 mA h g^−1^. The irreversible transformation into the Li_*x*_Mn_2_O_4_ spinel is thought to be accelerated, compared to the measurements at 25 °C.

The *E* of LMO (12 GPa) slowly increases from an open circuit voltage of approximately 3.0 V to ∼4.4 V with a *Q*_cha_ of ∼40 mA h g^−1^ during the initial charge. Afterwards, the *E* remains flat until finally reaching the cut-off voltage of 4.8 V. In contrast to the situation observed at 25 °C, the *Q*_cha_ increases dramatically to 185 mA h g^−1^, in comparison to the *Q*_theo_ of 65%. The redox reaction at approximately 4.4 V is not observed in LMO (5 GPa), whereas it is clearly seen in the LMO (8 GPa) (see Fig. S2a and b†). According to the first-principle calculations and numerical simulations reported by Ceder's group,^39–42^ the Li^+^ ion diffusion barrier for γ-LiFeO_2_-type structured materials is higher than that for other positive electrode materials such as LiCoO_2_ with an α-NaFeO_2_-type structure and LiMn_2_O_4_ with a spinel-type structure. Moreover, the Mn ions that occupied 0.8% of the 4*a* lithium sites (Table 3) would block the lithium diffusion path at 25 °C, as reported for LiFePO_4_, which has a one-dimensional lithium ion path along the *b* axis.^43^ Therefore, by increasing the temperature up to 40 °C, Li^+^ ions could be kinetically removed from the lattice during the initial charge reaction. Moreover, the initial charge curve exhibited an *E*_ave_ of 4.56 V, indicating that the Mn^3+^/Mn^4+^ redox potential in t-LiMnO_2_ was superior to that of o-LiMnO_2_ (*E*_ave_ = 3.86 V). Since the *W* for positive electrode materials is calculated as a product of the *Q*_recha_ and *E*_ave_, the *W* for t-LiMnO_2_ during the initial charge was estimated to be 844 mW h g^−1^ using a *Q*_cha_ = 185 mA h g^−1^ and *E*_ave_ = 4.56 V. This value was much larger than the *W* for LiMn_2_O_4_ (∼500 mW h g^−1^) which is already commercially available as the positive electrode material.^8^ A high *E*_ave_ is favorable for the potential application of t-LiMnO_2_ in this field.

The initial discharge curve, by contrast, could not maintain this *E*_ave_ and exhibited a *Q*_dis_ of 87 mA h g^−1^, resulting in a *W* value of 273 mW h g^−1^. This was due to the type of polarization, which was similar to that seen in the results obtained at 25 °C. With an increasing cycle number, the *Q*_cha_ and *Q*_dis_ improved to approximately 210 mA h g^−1^. Nevertheless, the *E*_ave_ was about 2.9 V at discharge and 4.0 V at charge over the course of more than two cycles. The *W* in the charge and discharge curves during the fifth cycle was 815 mW h g^−1^ and 618 mW h g^−1^, respectively.

### Crystal structure change upon electrochemical cycling

**Fig. 7:**
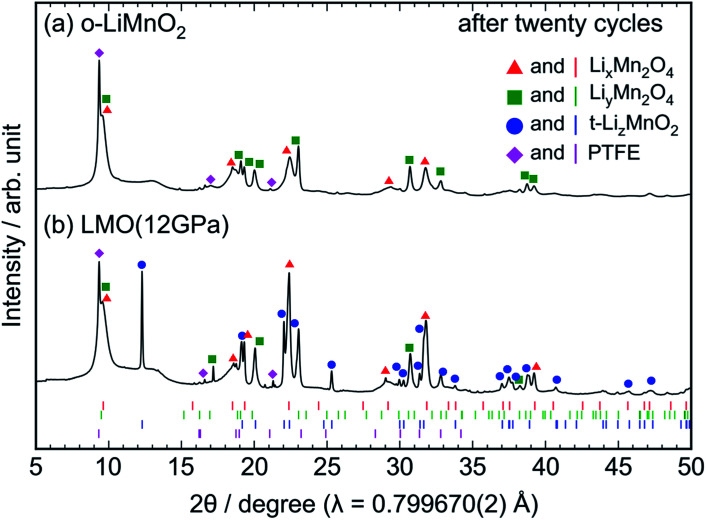
*Ex situ* XRD patterns of the cycled (a) o-LiMnO_2_ and (b) LMO (12 GPa) samples. The measurements were performed at the discharged states, as indicated by the red arrows in Fig. 5a and d. Diffraction lines represented by red triangles, green squares, blue circles, and magenta diamonds, originate from those from Li_*x*_Mn_2_O_4_ with the spinel structure, Li_*y*_Mn_2_O_4_ with tetragonal structure, t-Li_*z*_MnO_2_, and PTFE, respectively. The Bragg positions for each of these phases are also shown in the bottom using identically colored vertical lines.

*Ex situ* XRD measurements and Raman spectroscopy were conducted on the cycled electrodes to understand the phase transformation of t-LiMnO_2_ during cycling. The XRD patterns of the cycled o-LiMnO_2_ and LMO (12 GPa) samples are shown in Fig. 7a and b, respectively. The XRD pattern of o-LiMnO_2_ is assigned as a mixture of the Li_*x*_Mn_2_O_4_ spinel (*Fd*3̄*m*) with *x* < 1*,* lithiated Li_*y*_Mn_2_O_4_ tetragonal (*I*4_1_/*amd*) phase with *y* > 1, and PTFE originated from the binder in the electrode. The lattice parameters, which were calculated using the least squares method with more than five non-overlapping diffraction lines, are found to be *a*_c_ = 8.224(9) Å for Li_*x*_Mn_2_O_4_, and *a*_t_ = 5.666(6) Å and *c*_t_ = 9.151(9) Å for Li_*y*_Mn_2_O_4_. Since these lattice parameters correspond to those for Li_0.98_Mn_2_O_4_ (*a*_c_ = 8.230 Å) and Li_1.82_Mn_2_O_4_ (*a*_t_ = 5.654 Å and *c*_t_ = 9.202 Å),^44^ the *x* and *y* values are estimated to be 0.98 and 1.82, respectively.

As seen in Fig. 7b, the XRD pattern of the cycled LMO (12 GPa) sample is assigned as a mixture of the Li_*x*_Mn_2_O_4_ spinel, the Li_*y*_Mn_2_O_4_ tetragonal, t-Li_*z*_MnO_2_, and PTFE. The evolution of the Li_*x*_Mn_2_O_4_ spinel and Li_*y*_Mn_2_O_4_ tetragonal phases confirms the transformation to the spinel structure from t-LiMnO_2_ during cycling, as in the case for o-LiMnO_2_. The lattice parameters were calculated to be *a*_c_ = 8.252(4) Å for Li_*x*_Mn_2_O_4_, *a*_t_ = 5.657(4) Å and *c*_t_ = 9.173(7) Å for Li_*y*_Mn_2_O_4_, and *a*_t_ = 4.1854(8) Å and *c*_t_ = 8.230(2) Å for t-Li_*z*_MnO_2_. Thus, the *x*, *y*, and *z* values were estimated to be 0.98, 1.82, and ∼1, respectively.

**Fig. 8:**
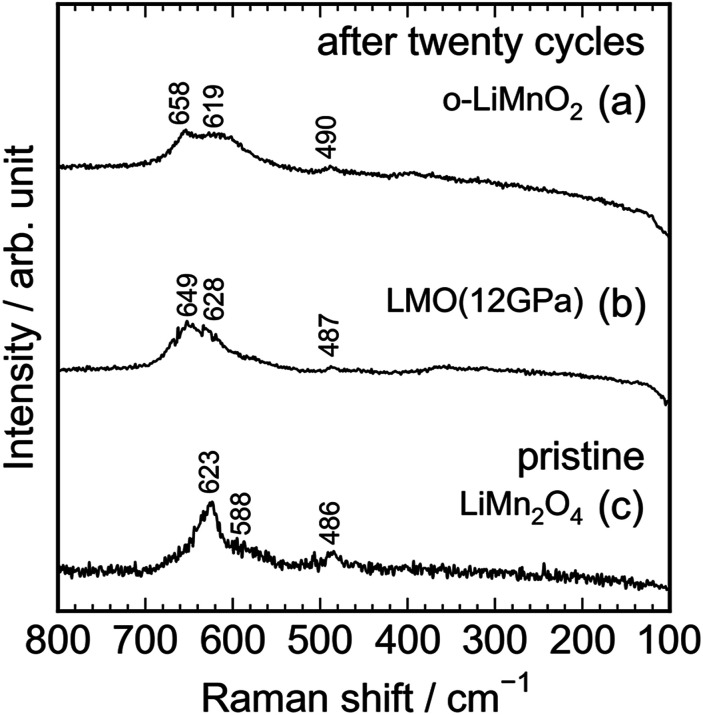
*Ex situ* Raman spectra of the cycled (a) o-LiMnO_2_ and (b) LMO (12 GPa) samples together with the Raman spectrum of the pristine (c) LiMn_2_O_4_. Raman spectra of (a) and (b) were taken at the discharged state, as indicated by the red arrows in Fig. 5a and d.

As seen in Fig. S3,† the Raman spectrum of the charged LMO (12 GPa) sample is similar with that of the pristine LMO (12 GPa) sample, except for the Raman band at 654 cm^−1^. This indicates that the local structure of LMO (12 GPa) is maintained during the initial charge reaction. However, Fig. 8 clarifies that the extended twenty cycle test converts the t-LiMnO_2_ structure into the spinel structure. That is, there are only three broad Raman bands at ∼650, 620, and 490 cm^−1^ in the cycled o-LiMnO_2_ and LMO (12 GPa) samples, and these Raman spectra are similar with the Raman spectrum of the pristine LiMn_2_O_4_ sample. The Raman bands at ∼650, 620, and 490 cm^−1^ are also observed in the cycled LMO (5 GPa) and LMO (8 GPa) samples (see Fig. S4†). It should be noted that the Raman spectra of the cycled (or charged) o-LiMnO_2_, LMO (5 GPa), LMO (8 GPa), and LMO (12 GPa) samples contain contributions of PTFE and AB, which were used for preparing the working electrodes. As seen in Fig. S5,† the Raman spectrum of PTFE shows three major Raman bands at 733, 385, and 290 cm^−1^, while that of AB is featureless. The contributions of PTFE and AB are, hence, negligibly small to the Raman spectra of the cycled (or charged) LMO samples.

**Fig. 9:**
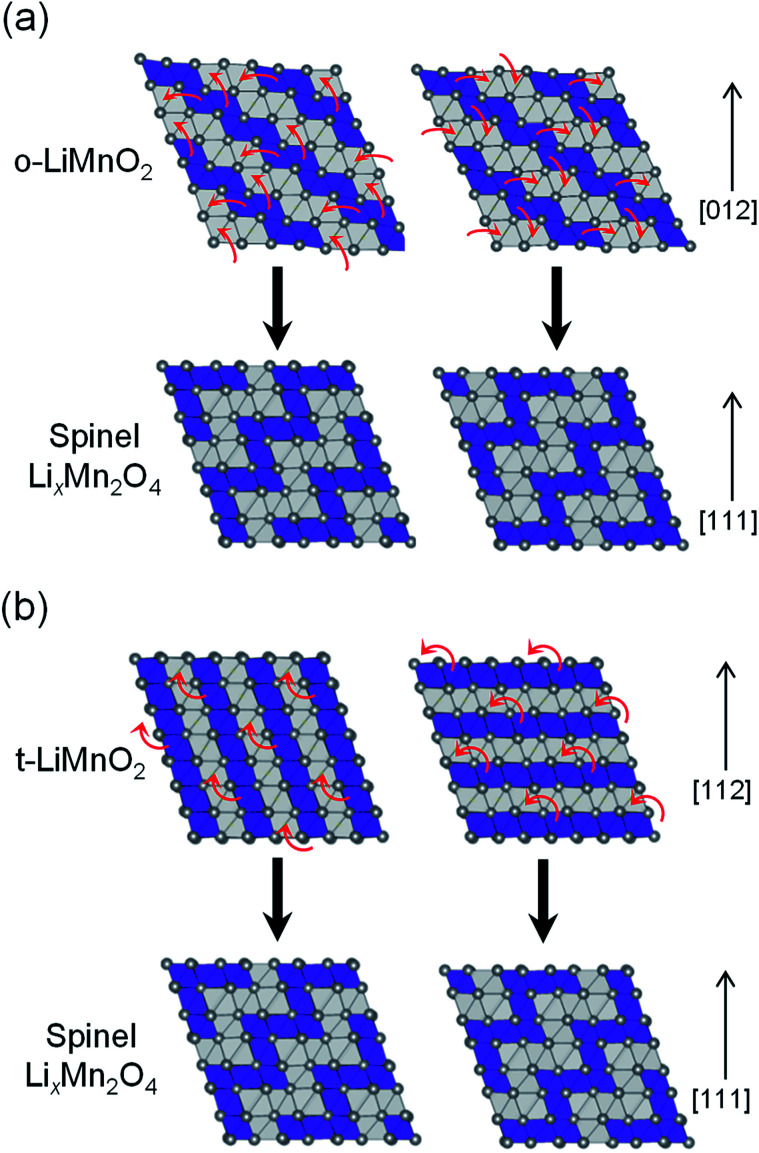
Schematics of the transformations into Li_*x*_Mn_2_O_4_ spinel: (a) from o-LiMnO_2_ and (b) from t-LiMnO_2_. The [012] in o-LiMnO_2_, [112] in t-LiMnO_2_, and [111] in Li_*x*_Mn_2_O_4_ directions correspond to those for oxygen closed-packing array. Purple and gray polyhedra are MnO_6_ and LiO_6_ (or O_6_: is vacancy) octahedra, respectively. Red arrows represent the Mn migration paths to vacant sites.

Fig. 9 shows possible mechanisms of the transformation into the spinel structure from the delithiated (a) o-LiMnO_2_ and (b) t-LiMnO_2_. Oxide ions in o-LiMnO_2_, t-LiMnO_2_, and Li_*x*_Mn_2_O_4_ spinel are arranged in the same ABCABC stacking manner along the [012] direction for o-LiMnO_2_, [112] for t-LiMnO_2_, and [111] for Li_*x*_Mn_2_O_4_, although the orderings between Mn and vacancy (or Li) octahedral sites are different each other. According to Thackeray *et al.*,^15^ Li_*x*_Mn_2_O_4_ is formed from two repeating types of MnO_2_ sheets, as shown in the left and right sides of Fig. 9. Thus, there are two ways for the transformation into the spinel from o-LiMnO_2_ or t-LiMnO_2_. In the case of o-LiMnO_2_, the transformation is caused by the displacement of half of the Mn ions to adjacent vacant octahedral sites.^15–17^ By contrast, the transformation of t-LiMnO_2_ is achieved by the displacement of a quarter of the Mn ions in the 4*b* sites into the vacant octahedral sites (4*a*) *via* adjacent octahedral sites without a rearrangement of the ABCABC oxygen packing (Fig. 9b). In this transformation, t-LiMnO_2_ has 50% lower amounts of migrated Mn ions, resulting in approximately twice longer in routes of Mn ions, compared to the case for o-LiMnO_2._

The transformation mechanism of t-LiMnO_2_ shows that a suppression of the Mn displacement at the charged state is essential for realizing electrochemical properties such as the *Q*_recha_, *E*_ave_, and *W* in the initial charge state over several cycles. Although the blocking effect of the movement of Mn ions in o-LiMnO_2_ has not been reported for the substitution of different cations with Li ions, a small amount of doping could be suitable for t-LiMnO_2_ due to the small amount of migrated Mn ions and their long displacement distance. Recently, electrochemical properties of tetragonal structured Li_0.35_MnO_2_(ref. 45) and Li_0.59_MnO_2_(ref. 46) nanoparticles were reported; *i.e.*, the initial *Q*_cha_ and *Q*_dis_ values of the nanosized Li_0.35_MnO_2_ are 72.9 and 178.8 mA h g^−1^, respectively. The *E* profile is similar to the discharge curve of the Li_*x*_Mn_2_O_4_ spinel, although the crystal structure of Li_0.35_MnO_2_ is maintained even after twenty cycles.^45^ Since the electrochemical properties and structural stabilities of t-LiMnO_2_ differ from those of Li_0.35_MnO_2_ (ref. 45) (or Li_0.59_MnO_2_),^47^ both Li content (*x*) and particle size also play important roles in stabilizing structure during cycling.

## Conclusions

We first investigated the electrochemical performances of t-LiMnO_2_ to obtain structural and electrochemical information regarding a series of LiMnO_2_ compounds with potential for the development of LMOs with high *W*. The t-LiMnO_2_ samples with γ-LiFeO_2_-type structure (*I*4_1_/*amd*) were prepared from o-LiMnO_2_ under pressures up to 8 GPa and at a temperature of 1000 °C, as evident by XRD measurements and Raman spectroscopy. Rietveld analyses indicated that the actual composition of t-LiMnO_2_ is Li_1.012_Mn_0.988_O_2_, in which Mn ions partially occupy the 4*a* lithium sites. The *Q*_cha_ of t-LiMnO_2_ rose to 185 mA h g^−1^ at 40 °C during the initial charge, even though it was only 37 mA h g^−1^ at 25 °C. Furthermore, the initial charge curve showed that the *E*_ave_ (4.56 V) with Mn^3+^/Mn^4+^ redox was the highest among LMOs such as o-LiMnO_2_ and LiMn_2_O_4_. However, the initial discharge curve did not maintain the *E*_ave_, and exhibited a *Q*_dis_ of 87 mA h g^−1^ due to an irreversible phase transformation into the Li_*x*_Mn_2_O_4_ spinel during the initial charge. Substitution of different cations and optimization of Li content and particle size will be necessary to maintain the electrochemical properties of t-LiMnO_2_ throughout long cycles.

## Conflicts of interest

There are no conflicts to declare.

## Supplementary Material

RA-008-C8RA03722A-s001
