# Supplementary material for: High-pressure synthesis and electrochemical properties of tetragonal LiMnO2
Source: RSC Adv. 2018 Jul 24;8(46):26325–34. doi: 10.1039/c8ra03722a (PMC9082863; doi:10.1039/c8ra03722a)
Supplement: RA-008-C8RA03722A-s001 [file RA-008-C8RA03722A-s001.pdf]

Electronic Supplementary Material (ESI) for RSC Advances.
This journal is © The Royal Society of Chemistry 2018

Supplementary Information for

High-pressure synthesis and electrochemical properties of tetragonal $LiMnO_2$

Takeshi Uyama[a*], Kazuhiko Mukai[a], and Ikuya Yamada[b]

[a] *Toyota Central Research and Development Laboratories Inc., Nagakute, Aichi 480–1192, Japan*

[b] *Department of Materials Science, Graduate School of Engineering, Osaka Prefecture University, 1–2 Gakuen, Sakai, Osaka 599–8590, Japan*

*Corresponding author

E-mail address: e1599@mosk.tytlabs.co.jp

Phone: +81-561-71-7202; Fax:+81-561-63-6119

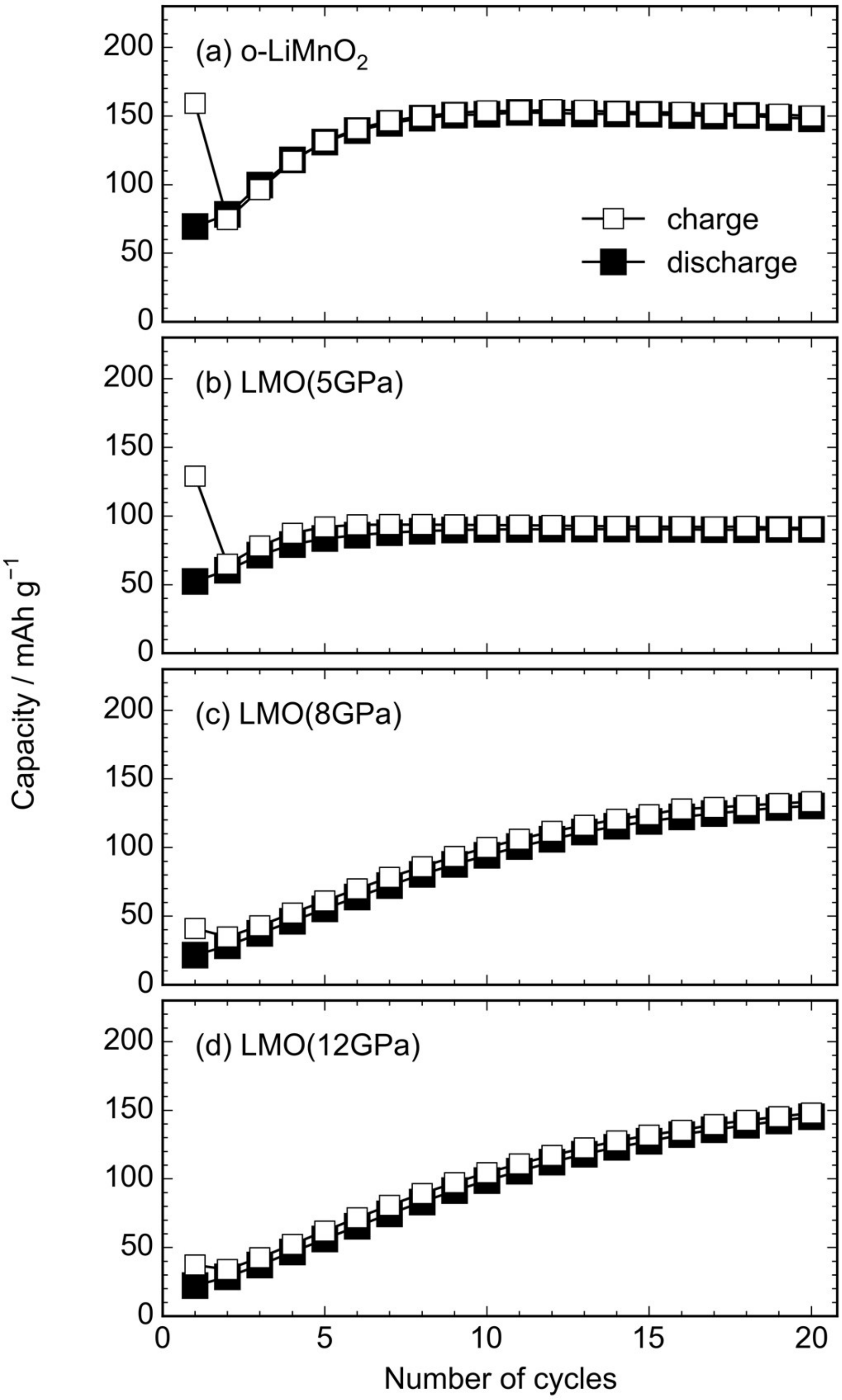

Fig. S1 Cycle performances at 25 °C of the (a) o-$LiMnO_2$, (b) LMO(5GPa), (c) LMO(8GPa), and (d) LMO(12GPa) samples. Open and closed squares indicate charge and discharge capacities, respectively.

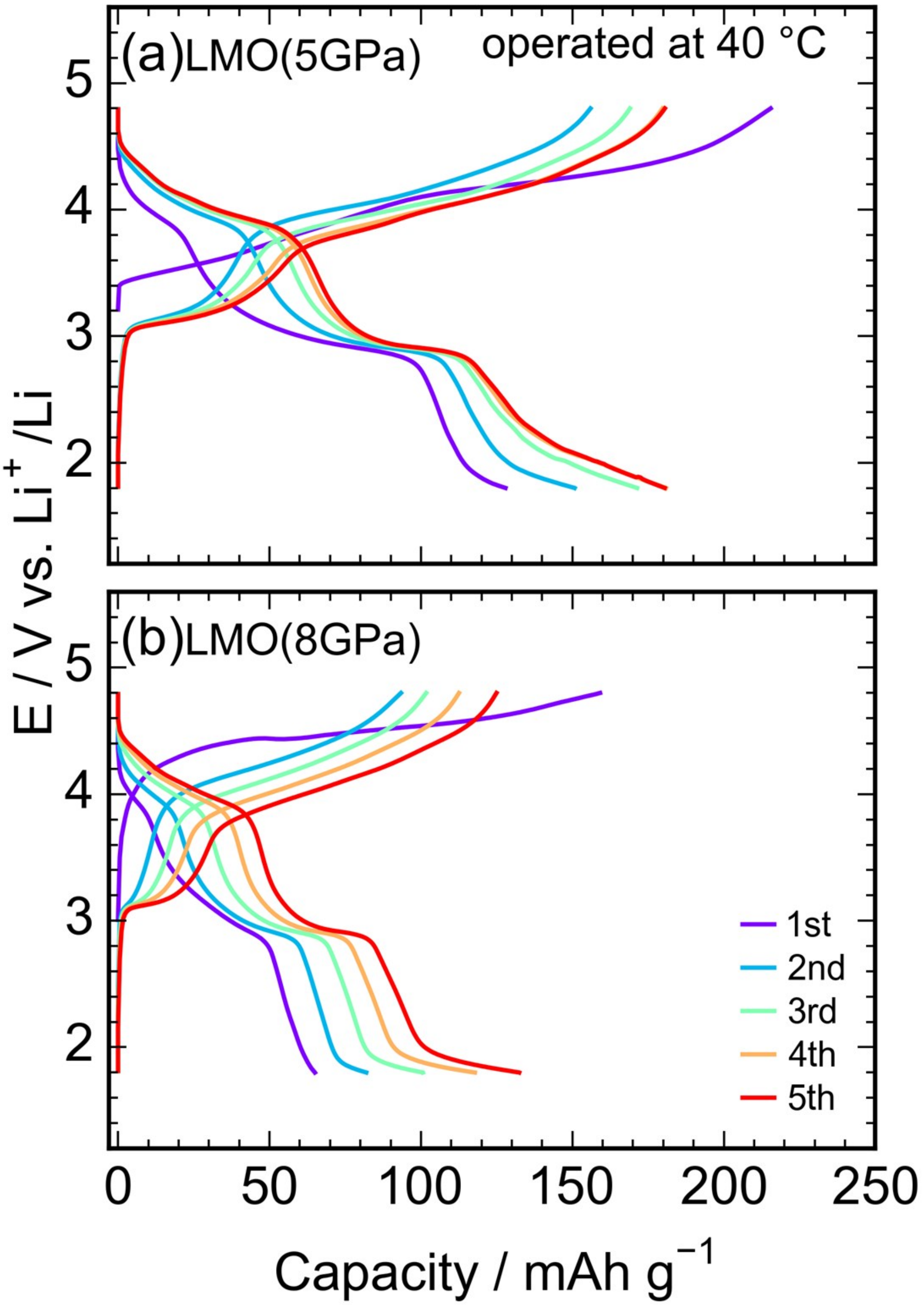

Fig. S2 Charge and discharge curves of the lithium cells with the (a) LMO(5GPa) and (b) LMO(8GPa) samples. The cells were operated at a temperature of 40 °C.

Table S1 Structure parameters of the LMO(5GPa) sample determined by the Rietveld analyses.

Phase	Space group	Atom	Wyckoff position	g^*	x	y	z^*	B^* / Å^2
o-$LiMnO_2$	*Pmmn*	Li1	2*b*	0.942(1)	1/4	3/4	0.127(2)	1.32
		Mn1	2*b*	0.058(1)	1/4	3/4	0.127(2)	1.32
		Mn2	2*b*	0.914(1)	1/4	3/4	0.634(1)	0.55
		Li2	2*b*	0.086(1)	1/4	3/4	0.634(1)	0.55
		O1	2*a*	1	1/4	1/4	0.151(1)	0.58
		O2	2*a*	1	1/4	1/4	0.608(1)	0.58
Composition : $Li_{1.028}Mn_{0.972}O_2$ (δ = 0.028), a_o = 2.80970(8) Å, b_o = 4.5766(1) Å, and c_o = 5.7504(1) Å.								
t-$LiMnO_2$	$I4_1/amd$	Li3	4*a*	0.976(7)	0	3/4	1/8	1.5(7)
		Mn3	4*a*	0.024(7)	0	3/4	1/8	1.5(7)
		Mn4	4*b*	0.948(7)	0	1/4	3/8	0.27(6)
		Li4	4*b*	0.052(7)	0	1/4	3/8	0.26(6)
		O3	8*e*	1	0	1/4	0.147(1)	1.1(2)
Composition : $Li_{1.028}Mn_{0.972}O_2$ (δ = 0.028), a_t = 4.1844(1) Å, and c_t = 8.2287(7) Å.								
Reliable factors : R_{wp} = 7.646 %, R_p = 5.273 %, and S = 0.939.								
Mass fractions : 86.6 wt% for o-$LiMnO_2$, 8.6 wt% for t-$LiMnO_2$, 4.0 wt% for Mn_3O_4, and 0.8 wt% for $LiMn_2O_4$.								

*Constraints : g(Mn1) = 1 − g(Li1), g(Mn2) = g(Li1) − δ, g(Li2) = 1 + δ − g(Li1), z(Mn1) = z(Li1), z(Li2) = z(Mn2), g(Mn3) = g(Li3), g(Mn4) = g(Li3) − δ, g(Li4) = 1 + δ − g(Li3), B(Mn3) = B(Li3), and B(Li4) = B(Mn4).

Table S2 Structure parameters of the LMO(8GPa) sample determined by the Rietveld analyses.

Phase	Space group	Atom	Wyckoff position	g^*	x	y	z	B^* / Å^2
t-$LiMnO_2$	$I4_1/amd$	Li1	4*a*	0.991(1)	0	3/4	1/8	1.44(8)
		Mn1	4*a*	0.009(1)	0	3/4	1/8	1.44(8)
		Mn2	4*b*	0.977(1)	0	1/4	3/8	0.28(1)
		Li2	4*b*	0.023(1)	0	1/4	3/8	0.28(1)
		O1	8*e*	1	0	1/4	0.141(1)	1.09(2)
Composition : $Li_{1.014}Mn_{0.986}O_2$ (δ = 0.014), a_t = 4.18259(2) Å, and c_t = 8.23443(6) Å.								
Reliable factors : R_{wp} = 6.076 %, R_p = 4.379 %, and S = 0.6781.								
Mass fractions : 96.4 wt% for t-$LiMnO_2$, 1.2 wt% for Mn_3O_4, and 2.3 wt% for $LiMn_2O_4$.								

*Constraints : g(Mn1) = 1 − g(Li1), g(Mn2) = g(Li1) − δ, g(Li2) = 1 + δ − g(Li1), B(Mn1) = B(Li1), and B(Li2) = B(Mn2).

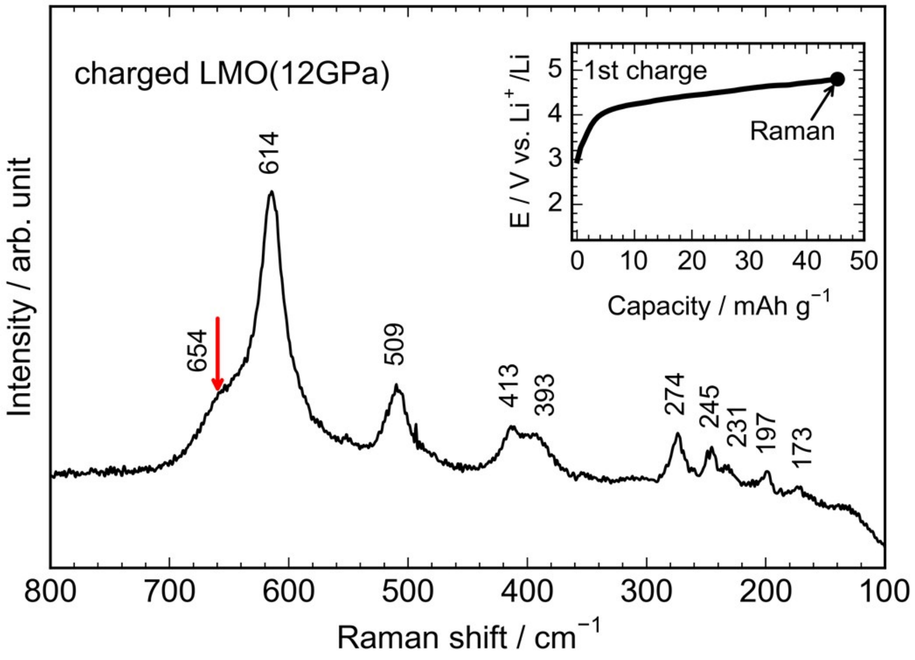

Fig. S3 *Ex situ* Raman spectrum of the charged LMO(12GPa) sample. The inset indicates its charge curve up to 4.8 V. The Raman band at 654 cm^{-1} is originated from $Li_xMn_2O_4$ spinel phase.

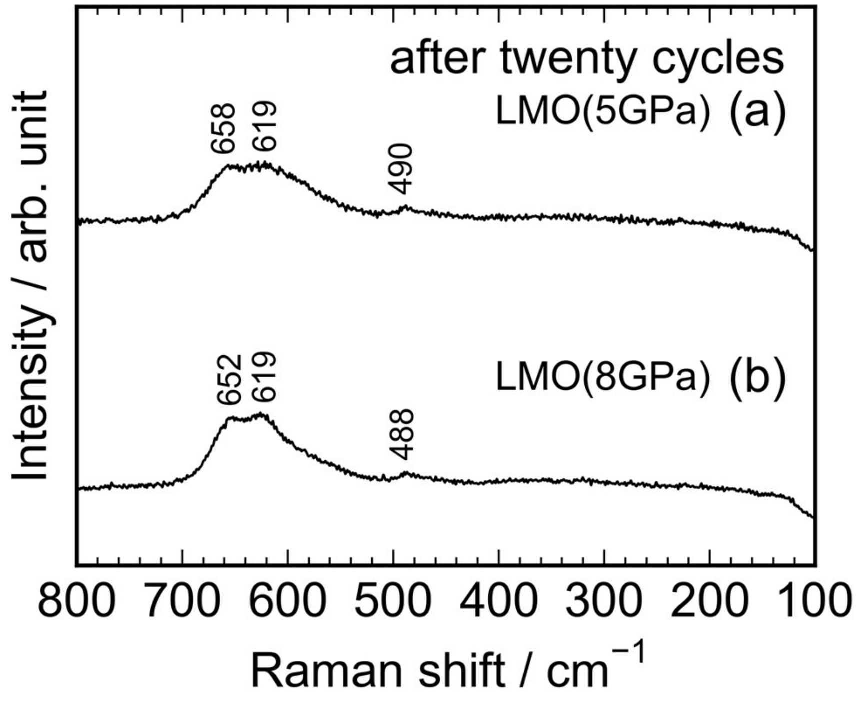

Fig. S4 *Ex situ* Raman spectra of the cycled (a) LMO(5GPa) and (b) LMO(8GPa) samples. Raman spectra were taken at the discharged state indicated by the red arrows in Figs. 5b and 5c in the text.

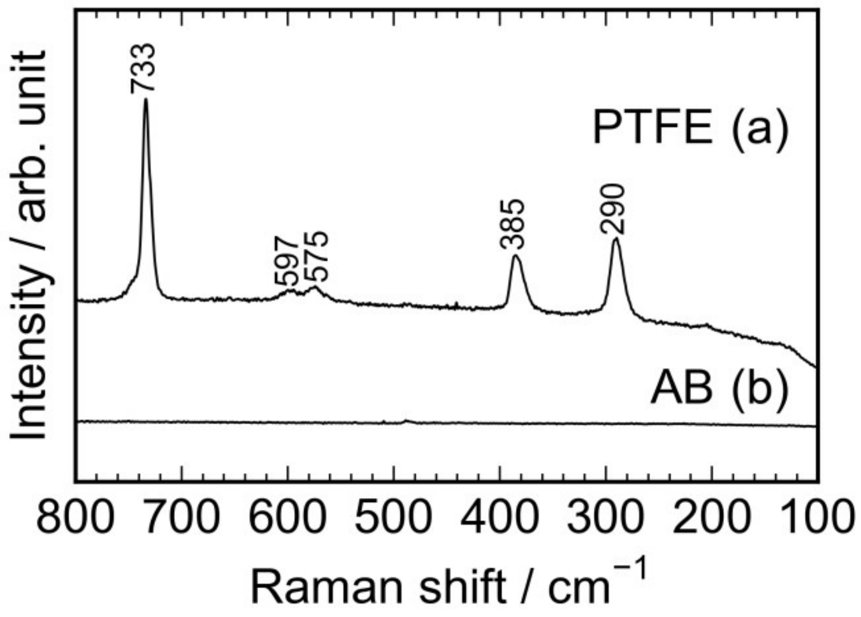

Fig. S5 Raman spectra for (a) PTFE and (b) AB.
